# Tumor microenvironment characterization in head and neck squamous carcinoma reveals distinct genomic alterations and clinical outcomes

**DOI:** 10.1002/ctm2.187

**Published:** 2020-09-27

**Authors:** Run Shi, Xuanwen Bao, Jing Sun, Shun Lu, Claus Belka, Minglun Li

**Affiliations:** ^1^ Department of Radiation Oncology University Hospital Munich Germany; ^2^ Technical University of Munich Munich Germany; ^3^ Department of Radiotherapy, Sichuan Cancer Hospital, School of Medicine University of Electronic Science and Technology of China Chengdu P. R. China

Dear Editor,

Head and neck squamous carcinoma (HNSC) is one of the most common malignancies worldwide. Despite recent advances in HNSC treatment, the prognosis remains unfavorable.^1^ Given the poor outcomes after standard treatment in HNSC, immunotherapy, such as anti‐PD‐1 therapy, is a promising alternative.^2^ However, due to the tumor heterogeneity and complexity of tumor microenvironment (TME),^3^, ^4^ immunotherapy benefits only a subset of HNSC patients, hence, the issue of patient selection becomes a critical challenge. Thus, a comprehensive understanding of HNSC needs to focus not only on tumor cells but also on TME. In this study, we aimed to characterize different TME landscapes by analyzing the infiltrating patterns of various TME cells, and to develop an individualized TME‐related scoring tool to predict cancer‐specific survival (CSS) in HNSC.

Normalized RNA‐seq data, somatic mutation data, and CSS information were obtained from The Cancer Genome Atlas (TCGA) portal (https://portal.gdc.cancer.gov/). xCell^5^ was used to quantify the abundance of immune and stroma cells involved in TME, and the immune, stroma, and TME scores of each sample were computed, respectively. The non‐negative matrix factorization (NMF) consensus clustering was used to identify different TME clusters. Principal coordinates analysis (PCoA) was used to visualize dissimilarity of different TME clusters based on their Bray–Curtis distance. Different immune responses were quantified using a single‐sample gene set enrichment analysis (ssGSEA)^6^ algorithm based on the transcriptome profiling data and corresponding gene sets retrieved from Molecular Signatures Database (MSigDB),^7^ and depicted by a radar chart with Z‐score normalization. ImmuCellAI^8^ was used to predict the response to immune checkpoint blockade (ICB) therapy. Using R package “maftools,” an oncoplot was generated to display the somatic mutation landscape in different TME clusters, and tumor mutational burden (TMB) was computed for each sample. The weighted gene coexpression network analysis (WGCNA)^9^ was used to identify a TME‐related gene module. Metascape^10^ was used to visualize the results of enrichment analysis. Subsequently, univariate and least absolute shrinkage and selection operator (LASSO) Cox regression analyses were step wisely performed to screen for the most robust prognostic genes. Finally, a formula which calculates TME‐related risk score (TMErs) was established to quantify the risk of cancer‐specific death: $TMErs=\sum_{i}\mathrm{LASSO}\;\mathrm{Cox}\;\mathrm{Coefficient}\left(\mathrm{mRN}A_{i}\right)\times \mathrm{Expression}\left(\mathrm{mRN}A_{i}\right).$ A total of 171 HNSC raw CEL files with CSS information produced from a same chip platform (Affymetrix HG‐U133 Plus 2.0) were downloaded from two Gene Expression Omnibus (GEO) datasets (GSE41613& GSE42743),and integrated to a singular cohort using a robust multiarray average (RMA) method, with Combat algorithm eliminating the batch effects. The prognostic capacity of TMErs was further validated in the independent GEO cohort. The Kaplan–Meier method was used to draw survival curves, and the log‐rank test was used to evaluate survival difference. The X‐tile software was used to select the most optimal cut points with the maximum of log‐rank statistics.^11^ Student's *t*‐test, one‐way ANOVA, or chi‐square test was used to evaluate the significance, and *P* value less than .05 was considered statistically significant.

Based on the abundance matrix of 36 cell types involved in TME, a fan phylogram was generated to show their similarity and distance (Figure 1A), and NMF consensus clustering was performed to divide 520 TCGA HNSC samples into three clusters (C1‐3) with an optimal *k* value of 3 (Figure 1B). A stacked barplot depicts the distinct patterns of the relative proportion of TME cells in the three identified clusters (Figure 1C). PCoA demonstrated that 520 samples were clearly separated into three distinct TME clusters (Figure 1D). Immune, stroma, and TME scores varied markedly among the three clusters (all, *P *< .001; Figure 1E‐G). Moreover, a radar chart demonstrated that the performances of different immune responses, such as humoral, adaptive, and innate immune responses, shrink progressively from C1 to C3 (Figure 1H). Accordingly, important inhibitory immune receptors and ligands, including PD‐1, PD‐L1, CTLA4, and TIGIT, were progressively downregulated from C1 to C3 (all, *P *< .001; Figure 1I). Furthermore, potential responses to ICB therapy differed among the three clusters (*P *= .002; Figure 1J). The Kaplan–Meier analysis showed that C1 exhibited best CSS, while C3 exhibited worst CSS (*P *= .008; Figure 1K). Landscape of somatic mutations was depicted in the three clusters (Figure 1L). Summarization of top mutated genes in each cluster showed the frequency of TP53 mutation in C1 is significantly lower than C2 and C3 (Figure 1M). In addition, significant difference of TMB was observed among the three clusters (*P *= .001; Figure 1N).

**FIGURE 1:**
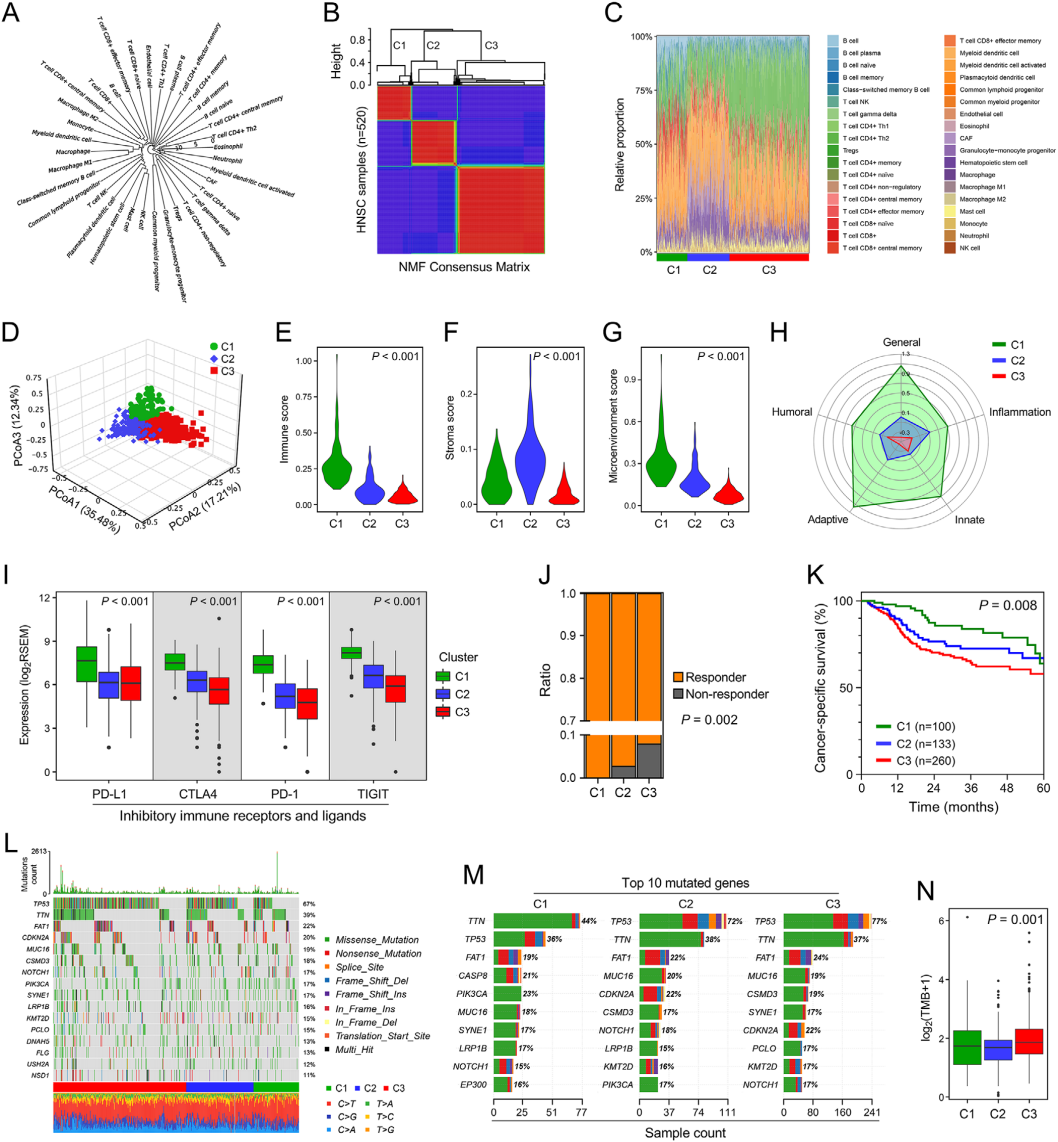
Different genomic alterations and clinical outcomes were observed in three NMF‐identified TME clusters. (A) A fan phylogram was generated to show the similarity and distance among the 36 cell types involved in TME. (B) NMF consensus clustering was performed to divide 520 TCGA HNSC samples into three clusters. (C) A stacked barplot depicts distinct TME patterns in the three clusters. (D) PCoA analysis demonstrated the three TME clusters were clearly separated. (E‐G) Immune, stroma, and TME scores varied markedly among the three TME clusters (all, *P *< .001). (H) A radar chart depicts the performances of different immune responses from C1 to C3. (I) Important inhibitory immune receptors and ligands, including PD‐1, PD‐L1, CTLA4, and TIGIT, were progressively downregulated from C1 to C3 (all, *P *< .001). (J) Potential responses to ICB therapy differed significantly among the three clusters (*P *= .002). (K) Different CSS was observed among the three clusters (*P *= .008). (L) Landscape of somatic mutations in the three clusters. (M) Summarization of 10 top mutated genes in each cluster. (N) Significant difference of TMB was observed in the three clusters (*P *= .001)

To quantify the risk assessment, we developed a TME‐related signature for CSS. The flowchart was presented in Figure 2A. With a power of 8 as the optimal soft threshold (Supporting information Figure S1), a total of 34 modules were generated using WGCNA, and the blue module was considered as “TME‐related module” due to its highest correlation with the TME score (*r* = .85, *P *= 3e‐140; Figure 2B).Gene Ontology enrichment analysis confirmed the blue module is involved in TME‐related biological processes (Figure 2C). Volcano plot shortlisted 141 promising candidates with a threshold *P* of univariate Cox regression analysis less than .001 (Figure 2D). Subsequently, LASSO Cox algorithm was used to identify the most robust prognostic genes. Tenfold cross‐validation was applied to overcome overfitting effect (Supporting information Figure S2), and an optimal λ of 0.0318 was selected (Figure 2E). Finally, nine genes remained, and the distribution of their LASSO Cox coefficients iss shown in Figure 2F. TMErs was calculated for each sample, and the entire cohort was divided into three parts: TMErs‐low, TMErs‐intermediate, and TMErs‐high groups. In the TCGA training cohort, HNSC patients in different TMErs groups exhibited significant difference in CSS (*P *< .001; Figure 2G), and the similar result was also observed in the GEO validation cohort (*P *< .001; Figure 2H). The results of log‐rank test between different TMErs groups in TCGA and GEO cohorts are summarized in Figure 2I.

**FIGURE 2:**
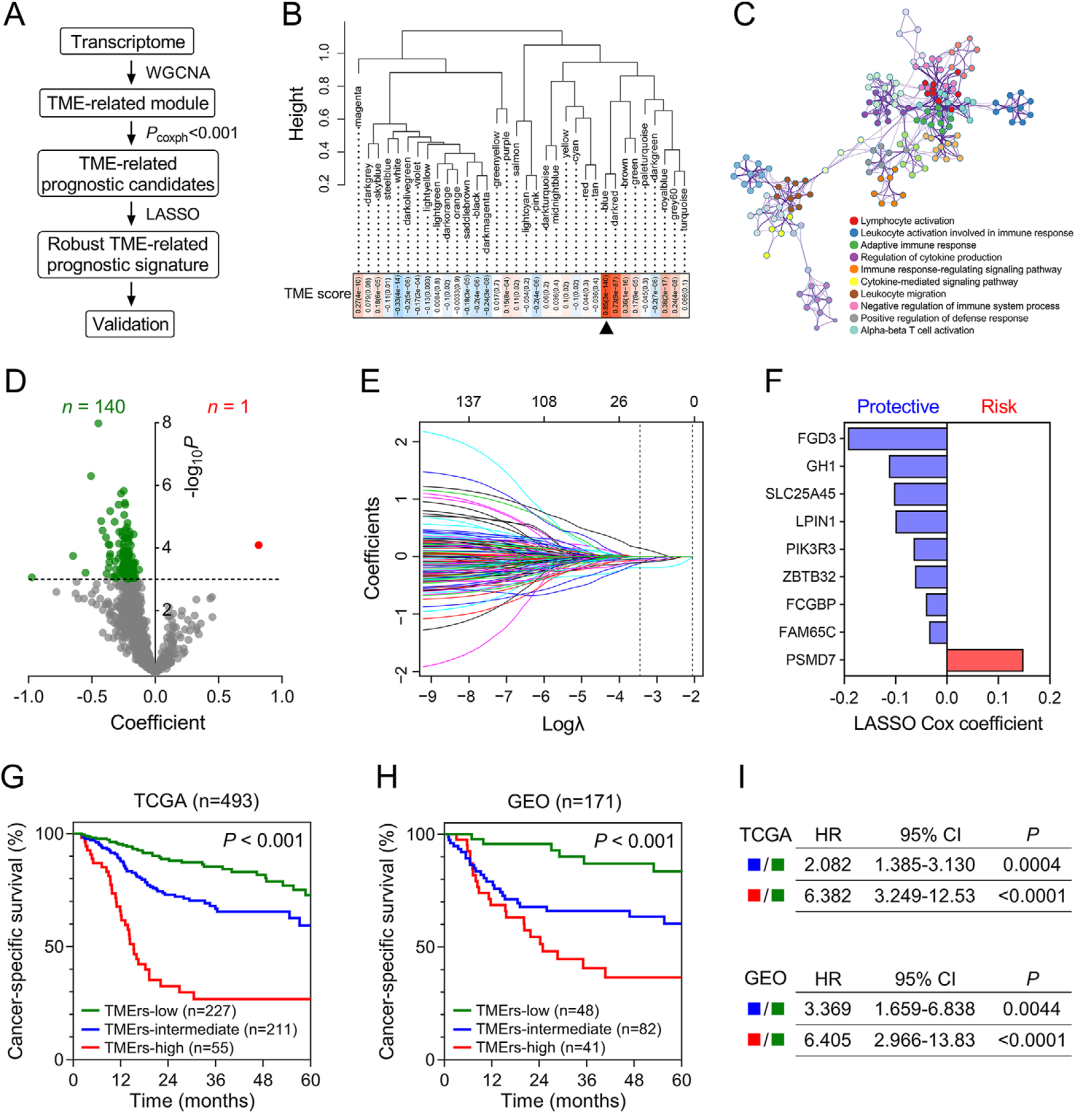
An individualized TME‐related prognostic signature was established and validated. (A) The flowchart. (B) A total of 34 modules were generated, and the blue module was selected due to its highest correlation with the TME score (*r* = 0.85, *P *= 3e‐140). (C) Gene Ontology enrichment analysis confirmed the blue module is involved in TME‐related biological processes. (D) Volcano plot shortlisted 141 promising candidates with a threshold *P* less than .001. (E) An optimal *λ* of 0.0318 was selected in the LASSO Cox algorithm. (F) LASSO Cox coefficients of the nine remaining genes. (G) In the TCGA training cohort, HNSC patients in different TMErs groups exhibited significant difference in CSS (*P *< .001), and (H) the similar result was also observed in the GEO validation cohort (*P *< .001). (I) Results of log‐rank tests between different TMErs groups in TCGA and GEO cohorts

In this study, we systematically evaluated the infiltrating abundance of various TME cells and compared clinical outcomes and genomic alterations in different TME clusters. Using NMF consensus clustering, 520 HNSC samples were divided into three distinct TME clusters. As well as TME score, potential response to ICB, CSS, and somatic mutation profile differed markedly among the three clusters. These findings demonstrated that the TME landscape could serve as a promising biomarker to discriminate high‐risk subset and guide patient selection for immunotherapy. To quantify the risk assessment, an individualized TME‐related prognostic signature was established, and further validated in an independent GEO HNSC cohort. The novelty of this study is the integration of TME gene coexpression network into the establishment of prognostic signature, which might increase the robustness of the present TMErs model.

The limitations should be acknowledged. First, this is a retrospective study based on public databases, thus, the sampling bias could not be completely excluded. Moreover, the sample size is relatively small. In summary, the clinical usefulness of TME should be further validated in a large prospective study.

## CONFLICT OF INTEREST

The authors declare no potential conflict of interest.

## Supporting information

Figure S1: A power of β = 8 was chosen as the optimal soft threshold to ensure a scale‐free co‐expression network in WGCNA.

Figure S2: In the LASSO Cox regression analysis, 10‐fold cross‐validation was applied to overcome over‐fitting effect.
