# Supplementary figures and images for: Tumor microenvironment characterization in head and neck squamous carcinoma reveals distinct genomic alterations and clinical outcomes
Source: Clin Transl Med. 2020 Sep 27;10(5):e187. doi: 10.1002/ctm2.187 (PMC7520081; doi:10.1002/ctm2.187)

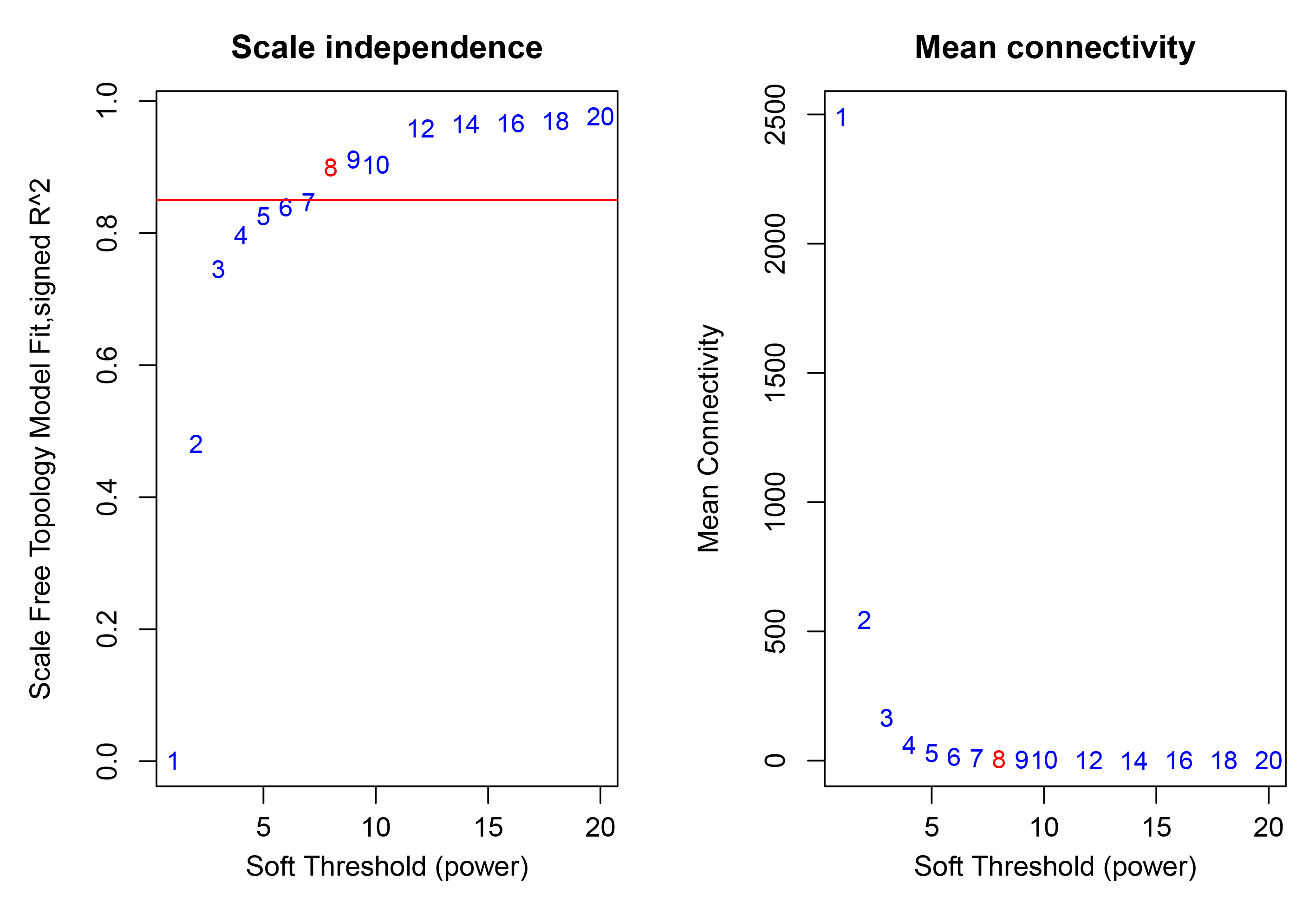

Supplement: Supplementary file 1 — Figure S1: A power of β = 8 was chosen as the optimal soft threshold to ensure a scale‐free co‐expression network in WGCNA. [file CTM2-10-e187-s001.tif]

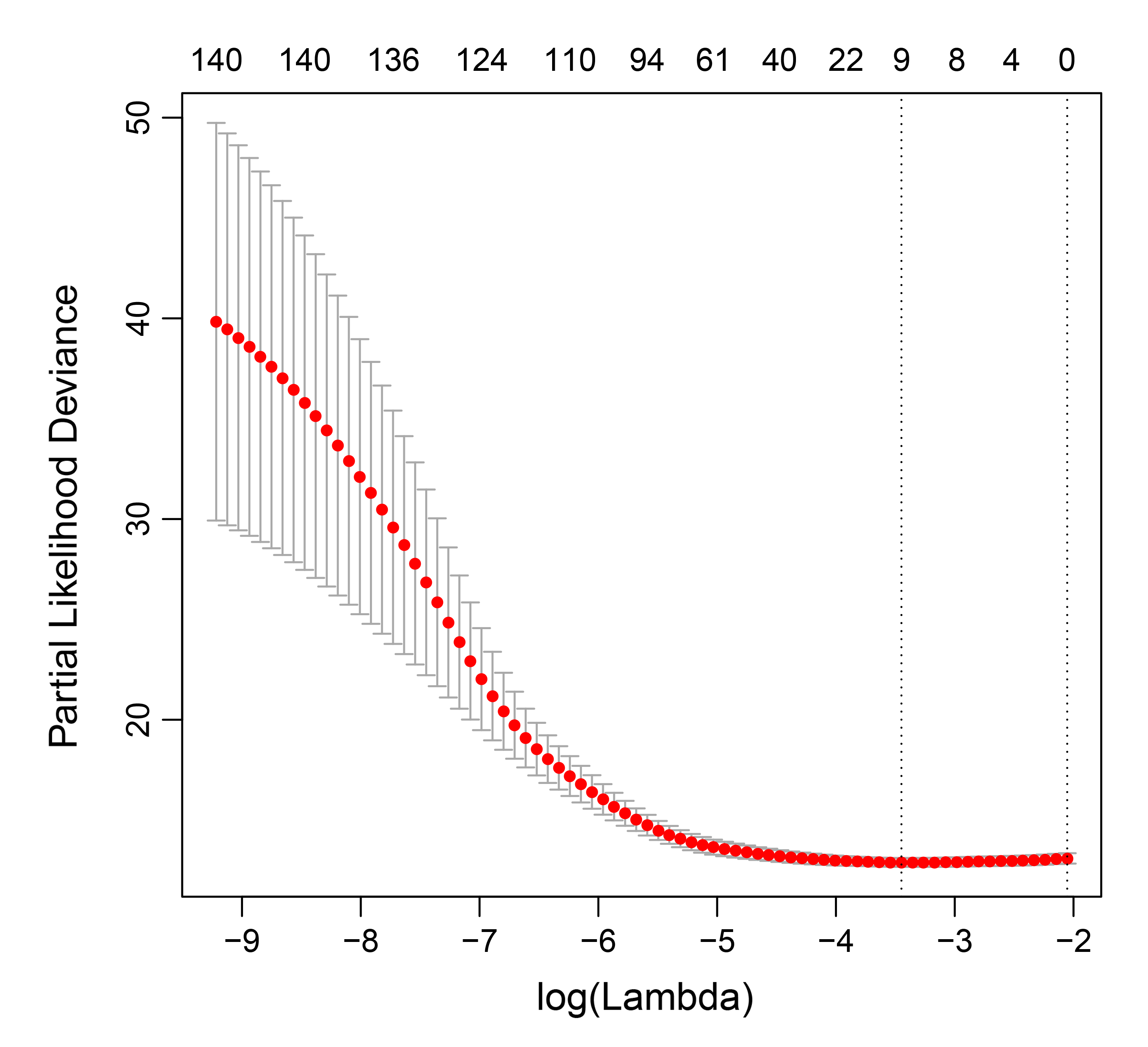

Supplement: Supplementary file 2 — Figure S2: In the LASSO Cox regression analysis, 10‐fold cross‐validation was applied to overcome over‐fitting effect. [file CTM2-10-e187-s002.tif]
